# Activity in dlPFC and its effective connectivity to vmPFC are associated with temporal discounting

**DOI:** 10.3389/fnins.2014.00050

**Published:** 2014-03-18

**Authors:** Todd A. Hare, Shabnam Hakimi, Antonio Rangel

**Affiliations:** ^1^Laboratory for Social and Neural Systems Research, Department of Economics, University of ZurichZurich, Switzerland; ^2^Division of Humanities and Social Science, California Institute of TechnologyPasadena, CA, USA; ^3^Computational and Neural Systems, California Institute of TechnologyPasadena, CA, USA

**Keywords:** fMRI, temporal discounting, vmPFC, DLPFC, effective connectivity, individual differences, delay of gratification

## Abstract

There is widespread interest in identifying computational and neurobiological mechanisms that influence the ability to choose long-term benefits over more proximal and readily available rewards in domains such as dietary and economic choice. We present the results of a human fMRI study that examines how neural activity relates to observed individual differences in the discounting of future rewards during an intertemporal monetary choice task. We found that a region of left dorsolateral prefrontal cortex (dlPFC) BA-46 was more active in trials where subjects chose delayed rewards, after controlling for the subjective value of those rewards. We also found that the connectivity from dlPFC BA-46 to a region of ventromedial prefrontal cortex (vmPFC) widely associated with the computation of stimulus values, increased at the time of choice, and especially during trials in which subjects chose delayed rewards. Finally, we found that estimates of effective connectivity between these two regions played a critical role in predicting out-of-sample, between-subject differences in discount rates. Together with previous findings in dietary choice, these results suggest that a common set of computational and neurobiological mechanisms facilitate choices in favor of long-term reward in both settings.

## Introduction

Impaired ability to delay gratification is thought to play a critical role in sub-optimal decision-making, and in conditions like addiction and obesity (Chambers et al., 2007; Monterosso and Ainslie, 2007; Peters and Buchel, 2011). As a result, there is a widespread, on-going effort to characterize the computational and neurobiological mechanisms underlying this form of self-control. Two types of paradigms have been widely used in behavioral neuroscience to examine these mechanisms. First, are tasks involving intertemporal decisions between rewards, often money, in which subjects choose between sooner-smaller amounts and later-larger ones (Rachlin, 2000; Ainslie, 2001; McClure et al., 2004; Kable and Glimcher, 2007; McClure et al., 2007; Ballard and Knutson, 2009; Gregorios-Pippas et al., 2009; Carter et al., 2010; Monterosso and Luo, 2010; Peters and Buchel, 2010, 2011; Luo et al., 2012). Second, are tasks involving dietary choices, in which subjects make choices between foods that vary in their tastiness and healthiness (Hare et al., 2009, 2011a; Volkow et al., 2011).

In previous work investigating dietary self-control, we found important commonalities and differences between successful and unsuccessful dieters (Hare et al., 2009). Behaviorally, the two groups differed on the relative weight that they placed on the health and taste attributes of foods in making their decisions (with successful dieters weighting both health and taste, and unsuccessful dieters weighting only taste). Neurally, the ventromedial prefrontal cortex (vmPFC) encoded the value of foods at the time of choice equally for both groups. The critical difference had to do with the role of left dorsolateral prefrontal cortex (dlPFC). In successful dieters, dlPFC came on-line and exhibited increased effective connectivity with vmPFC during choices that required self-control (e.g., refusing to eat tasty, but unhealthy candy). In contrast, unsuccessful dieters did not exhibit this pattern of connectivity. Furthermore, in a subsequent study we found that non-dieting participants behaved like successful dieters if they were given an exogenous reminder to pay attention to health information, and that the reminder activated the same dlPFC-vmPFC networks that successful dieters activated on their own (Hare et al., 2011a).

These findings led us to propose the following model of the computational and neurobiological processes at work in self-control (Hare et al., 2009, 2011a,b; Rangel and Hare, 2010; Harris et al., 2013). In the model, the vmPFC computes the value of options at the time of decision, by first assessing their various attributes, and then integrating them into a net value for the option as a whole. Importantly, “basic” attributes like tastiness might always be represented in the final value. However, more abstract attributes like healthiness are only represented, or are represented more strongly, if the dlPFC comes online and modulates activity in vmPFC so that its value computations incorporate them. This modulation is critical for optimal decision-making because, if some of the attributes are not represented or weighted properly, the vmPFC will assign values to options that are not consistent with the long-term, goal-relevant (e.g., proper nutrition) rewards that they generate.

An important open question is whether this model is also at work in other decision domains, such as those involving intertemporal monetary tradeoffs. This question is important because comparing the mechanisms at work in different decision contexts is a critical step in identifying common mechanisms that facilitate self-control. Theoretically, these circuits should also influence the degree of discounting for delayed rewards in the case of intertemporal choice, as long as dlPFC modulation of vmPFC can lead to an increased (or decreased) weighting for delayed rewards.

Here we address this open question by testing the following three hypotheses. First, we hypothesized that regions of left dlPFC similar to those that are more active during self-control in dietary choice would also be more active in intertemporal choice when the subjects choose the larger-delayed payment over the money available today, after controlling for their relative subjective values. Note that it is crucial to control for the subjective values because, if the subjective value of the delayed reward is large enough, the decision to wait becomes trivial. Second, we hypothesized that effective connectivity from left dlPFC to vmPFC would be stronger during trials in which subjects choose larger-delayed rewards (again controlling for subjective value), which is consistent with the idea that dlPFC can modulate the value signals in vmPFC so that they place more weight on the value of delayed payouts. Third, we hypothesized that the levels of activation in dlPFC, as well as its effective connectivity to vmPFC, would help to explain differences in discount rates across subjects.

These hypotheses are based not only on previous work in dietary choice, but also on findings from the previous literature on goal-directed choice. First, areas of vmPFC have consistently been shown to correlate with stimulus values at the time of choice across a wide variety of decision contexts (Tom et al., 2007; Boorman et al., 2009; Lebreton et al., 2009; Basten et al., 2010; Hare et al., 2010; Plassmann et al., 2010; Shenhav and Greene, 2010; Clithero et al., 2011; Kahnt et al., 2011; Park et al., 2011), including decisions involving intertemporal tradeoffs (Kable and Glimcher, 2007; Ballard and Knutson, 2009; Hare et al., 2009, 2011a; Carter et al., 2010; Peters and Buchel, 2010). Second, previous studies have associated responses in left dlPFC with choosing to wait for delayed monetary rewards using transcranial magnetic stimulation (TMS) and fMRI (McClure et al., 2004; Figner et al., 2010; Luo et al., 2012). In particular, Figner et al. (2010) showed that temporarily reducing activity in left dlPFC via TMS results in subjects making more impatient choices, thus, establishing a causal role for this region in temporal discounting. Third, recent studies have found that resting-state connectivity in networks including left dlPFC was correlated with discount rates (Gianotti et al., 2012; Li et al., 2013).

Despite the attractiveness of the theory, and the body of consistent evidence, critical questions remain open. In particular, none of the previous studies have examined the effective connectivity between dlPFC and vmPFC during intertemporal monetary choices, nor have they established that the dlPFC influences discount rates through a mechanism that involves the modulation of the stimulus values computed in vmPFC, or that the effective connectivity runs from dlPFC to vmPFC, and not the other way around. Here we are able to address these questions by estimating Dynamic Causal Models (Friston et al., 2003), and using those parameter estimates to explain and predict differences in discount rates across individuals.

## Materials and methods

### Participants

Twenty-seven subjects (18 males; age: mean = 24.1 years; range = 19–40) were included in the study. Two additional subjects were excluded because of excessive head motion during the scanning session (>2 mm in translation or rotation). All participants had normal or corrected-to-normal vision, no history of neurological, psychiatric, or metabolic illness, and were not taking any medications that interfere with the blood oxygenation level-dependent (BOLD) signal at the time of scanning. The Institutional Review Board at California Institute of Technology approved the methods and procedures used in this study.

### Intertemporal choice (ITC) task

On every trial, subjects chose between getting $25 at the end of the experiment, or getting an equal or larger amount at a later date. The later offers ranged from $25 to $54; with a delay from 7 to 200 days. Subjects made 216 decisions. The unique combinations of amount and delay used are shown in Table 1. All subjects saw the same set of options, although in different random orders. Each option was shown twice. Note that by presenting all subjects with the same options we were able to control for the objective reward levels when testing how neural activity relates to discount rates. Although beneficial for the hypotheses tested in previous studies (Kable and Glimcher, 2007, 2010; Peters and Buchel, 2009, 2010), tailoring the choice sets around the indifference points of each subject would create a confound with objective value when examining how individual differences in neural activity relate to discount rates because less patient subjects would be shown delayed rewards with higher monetary values.

**Table 1:** **Amounts by delay**.

**Delay**	**Amount**					
7	25	26	28	30	32	35
10	25	26	27	29	30	32
12	25	26	28	31	33	35
14	25	26	28	32	35	39
21	26	27	29	30	32	38
25	27	29	31	33	35	46
28	26	28	32	35	39	46
30	26	27	29	30	32	38
40	27	33	35	40	47	54
45	26	29	31	35	40	46
50	27	30	35	40	46	54
60	29	33	35	40	47	54
90	26	30	33	40	46	54
95	31	33	35	40	47	54
100	26	31	38	39	46	54
150	31	33	35	40	47	54
180	27	31	35	39	46	54
200	26	28	35	39	47	54

*Delays are listed in days and amounts in USD. The combinations of amount and delay were chosen to facilitate the estimation of hyperbolic discounting parameters within the range 0.0005–0.05 that is commonly reported in monetary discounting tasks*.

**Figure 1:**
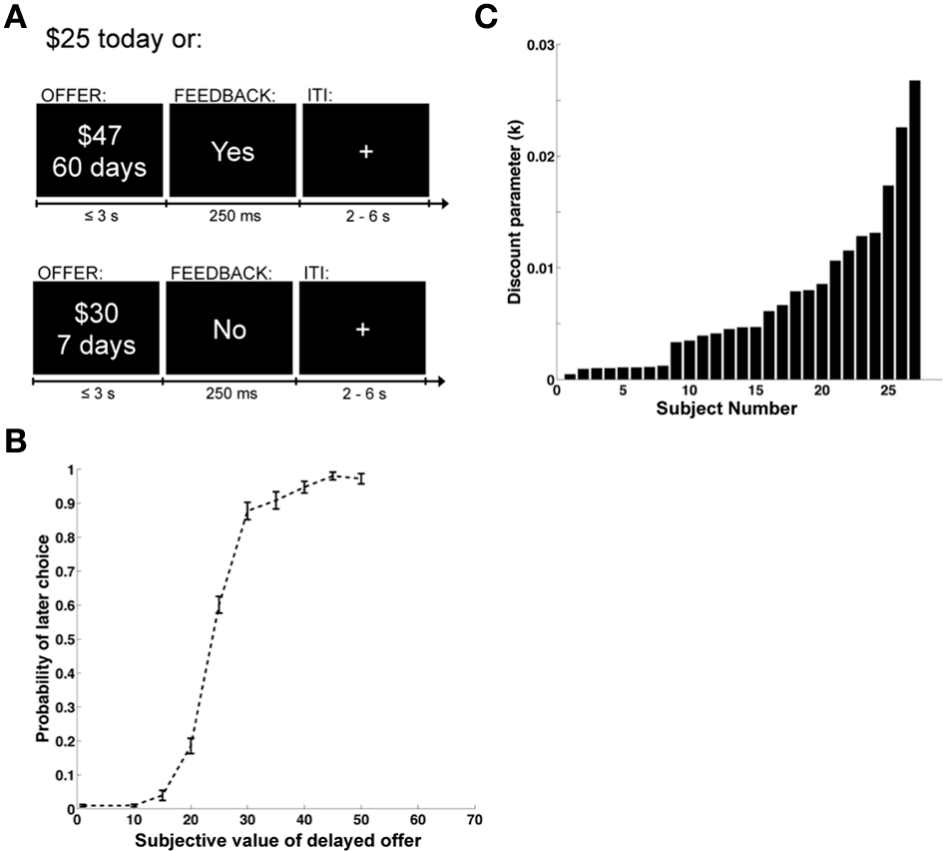
**Task design and behavioral data**. **(A)** Example display screens and timing parameters. **(B)** Choice curve displaying the probability of choosing the larger, delayed reward. The y-axis shows the probability of selecting the future reward and the x-axis displays the stimulus value of the future reward. Error bars represent the standard error of the mean. **(C)** Bar graph showing the distribution of discounting parameters across subjects. The x-axis represents individual subjects and the y-axis is the magnitude of the discount parameter k from a hyperbolic discounting function.

As described in Figure 1A, each trial began with an offer presented onscreen. Participants were required to press within 3 s to indicate whether or not they accepted the delayed reward offered. Only the varying delayed option was presented onscreen. A button press response resulted in the termination of the offer screen, and the appearance of a feedback screen for 250 ms displaying “Yes,” if the delayed offer was accepted, or “No,” if it was rejected. The phrase “No decision received” was displayed if the subject failed to respond within 3 s (mean = 2% of trials, standard deviation 5%, median = 0%). Trials were separated by a fixation cross of random duration (uniform: 2–6 s). The assignment of left/right button presses to accept/reject responses was counterbalanced across subjects. At the end of the experiment a single trial was randomly chosen and implemented: subjects received the chosen option in addition to $50 (available immediately) for participating in the study. All payments were made using prepaid debit cards given to the subjects at the end of the experiment. This allowed us to make the delayed payments available on the appropriate date, without requiring subjects to return to the lab.

### Behavioral data analysis

We estimated an individual discount factor (denoted by *k*) for each subject using maximum likelihood. In particular, we assumed that subjects assigned value to the delayed options using a hyperbolic discounting function, in which the value of $*A* with a delay of *D* days is given by

dSV=A/(1+kD),

where *dSV* denotes the discounted stimulus value. We also assumed that the probability of accepting the delayed option is given by the soft-max function

$$P(\text{Yes})={\left(1+exp(b*(25-dSV))\right)}^{-1},$$

where *b* is a non-negative parameter that modulates the slope of the psychometric choice function. Note that in this formula the value of the constant reference option is $25.

### Imaging data acquisition

FMRI data were collected in a Siemens (Erlangen, Germany) 3.0 Tesla Trio MRI scanner. Using an eight-channel, phased array head coil, we collected gradient echo T2^*^-weighted echoplanar (EPI) images with BOLD contrast. In order to optimize BOLD sensitivity, we used a tilted acquisition in an orientation 30° oblique to the anterior-posterior commissure line (Deichmann et al., 2003). The imaging parameters were as follows: *TR* = 2500 ms; *TE* = 30 ms; flip angle = 80°; FOV = 192 mm; in-plane resolution = 3 × 3-mm; and 40 3-mm slices (0.3-mm gap) with ascending acquisition. While in the scanner, subjects completed two runs of the ITC task (with 323 volumes acquired per run). They also completed an additional task involving the degustation of liquid rewards that is not relevant to this study (task order was counterbalanced across participants). High-resolution, whole-brain T1-weighted structural images (*TR* = 1500 ms; *TE* = 3.05 ms; flip angle = 10°; voxel resolution = 1 mm^3^; single-shot, ascending acquisition) were also collected for each participant. These images were co-registered with the their respective EPI images to assist with the anatomical localization of the functional activations.

### fMRI data preprocessing

Imaging data were preprocessed using SPM8 (Wellcome Department of Imaging Neuroscience, Institute of Neurology, London, UK). Data were corrected for motion with realignment to the mean image, spatially normalized to the Montreal Neurological Institute EPI template, resampled to 3 mm^3^ voxels, and spatially smoothed using a Gaussian kernel (full-width-at-half-maximum = 8 mm). Data were also temporally filtered using a filter width of 128 s.

### GLMs

We estimated two different mixed effect models of the BOLD responses, with first order auto correlation correction [AR(1)]. The models were designed to localize in our sample the areas of vmPFC that, as discussed in the Introduction, have been repeatedly shown to correlate with stimulus values at the time of choice. The models are identical except for the specification of the value modulators.

The first model, GLM-dSV, had the following regressors of interest: (1) An indicator function beginning at the onset of each decision screen with duration equal to the reaction time for that trial, (2) the indicator function modulated by the subject specific value of each delayed offer (*dSV*), and (3) the indicator function modulated by the variable *Accept* (which equals 1 if the subject chooses the delayed outcome, and zero otherwise). The third regressor was orthogonalized with respect to the second one in order to assign any shared variance between them to the *dSV* regressor. The model also included session dummies, linear time trends, and head movements as regressors of no interest.

The second model, GLM-rdSV, was identical except for the specification of the parametric regressor. In particular, for the reasons described in the Results section, we defined a relative discounted subjective value (*rdSV*) variable, which is equal to *dSV* −25 for subjects that choose the delayed variable more than 50% of the time (15 subjects), and is equal to 25-*dSV* for those that choose the immediate option more frequently (12 subjects).

Both GLMs were estimated in three steps. First we estimated the model at the individual level. Second, we calculated the following first-level single-subject contrasts: regressor 2 (d*SV* or rd*SV*) vs. baseline, and regressor 3 (*Accept*) vs. baseline. Third, we calculated second-level group contrasts using one-sample *t*-tests on the single-subject contrasts.

We controlled for multiple comparisons at the cluster level using an individual voxel threshold of *p* < 0.005 to achieve a whole brain corrected (WBC) *p*-values less than 0.05 (cluster sizes are listed in each table). We also used small volume corrections (SVCs) in areas of a priori interest to the study of the self-control mechanisms that are at the core of the hypotheses tested here. We carried out an SVC in the vmPFC using an anatomical mask based on the AAL atlas (Tzourio-Mazoyer et al., 2002) that included the rectal gyrus, medial orbitofrontal, and anterior cingulate cortex below *z* = 5 (1619 3-mm^3^ voxels). A region in left dorsolateral PFC, in Brodmann Area (BA) 46, has been shown to play a role in various types of self-control tasks (Hare et al., 2009, 2011a; Figner et al., 2010). Because anatomical masks of dlPFC lacked the required specificity to isolate this region, we carried out SVC using a mask composed of a 10-mm radius sphere centered around the target coordinates (*x, y, z* = −36, 30, 27) used by Figner et al. (2010) to demonstrate a causal role of dlPFC on discounting behavior. The TMS stimulation of left dlPFC in this study was directed to the F3 location of the international 10–20 system for electrode placement, and we used the transformation algorithms in the Münster T2T-Converter software (http://wwwneuro03.uni-muenster.de/ger/t2tconv/) to compute an estimate of the underlying MNI coordinates.

To assess the impact of the *dSV* regressor on model fit within the reward valuation network identified in two recent meta-analyses (Bartra et al., 2013; Clithero and Rangel, 2013), we re-estimated GLM-dSV and a reduced form of GLM-dSV using the Variational Bayes routines in SPM8 (Penny et al., 2003, 2005). The reduced form of GLM-dSV excluded the parametric regressor for *dSV*, but included all other regressors described above. Following estimation of both models at the single subject level, we used a random effects Bayesian model selection (BMS) procedure (Rosa et al., 2010) to assess the variance explained by the *dSV* regressor independent of the sign (+/−) on its coefficient in a fashion similar to an *F*-test, but additionally accounting for model complexity. This BMS procedure generated exceedance probabilities from the model evidence for each GLM. The exceedance probabilities correspond to the belief that the full version of GLM-dSV is more likely than the reduced version given the data from all subjects (or vice versa). We evaluated the exceedance probability in all voxels within the conjunction of masks generated from recent meta-analyses on reward valuation. Specifically, we formed the conjunction mask from the voxels shown in Clithero and Rangel's (2013) Figure 3 and Bartra et al. (2013) Figure 3A. This mask included voxels in vmPFC, ventral striatum (vStr), and posterior cingulate cortex (PCC) consistently found to positively correlate with the value of reward across reward types and decision contexts in the meta-analyses listed above. This mask can be downloaded from the following website: http://www.rnl.caltech.edu/resources/index.html.

### Dynamic causal modeling (DCM)

We tested the hypothesis that the effective connectivity from left dlPFC-BA46 to vmPFC plays a critical role in self-control using DCM (Friston et al., 2003). The analysis proceeded in several steps.

First, for each subject we extracted average activation time courses from vmPFC and left dlPFC-BA46. In particular, for every subject we defined an ROI with a 5-mm radius, and a center given by each subject's most significant voxel within the group ROIs. The group ROI in vmPFC was defined based on the conjunction between voxels showing an effect for the *rdSV* and *Accept* regressors from GLM-rdSV at an uncorrected threshold of *p* < 0.005. The group ROI in dlPFC-BA 46 was defined as all voxels showing an effect for the *Accept* regressor from GLM-rdSV at an uncorrected threshold of *p* < 0.005.

Second, we optimized the basic architecture of the DCM, in terms of where experimental inputs entered. To do so, we estimated 64 different DCMs that could be organized into four different families (Figure 3A), based on how the variables *rdSV* and *Accept* affect activity in dlPFC and vmPFC. Each family contained 16 models that varied in terms of the combinations of connectivity between vmPFC and dlPFC-BA46 as a function of three events: fixation, all choice periods, and choice periods in which the delayed option is selected.

Third, we compared each model family using BMS (Stephan et al., 2009) to determine the most likely pattern of task related inputs into dlPFC and vmPFC.

Fourth, having optimized the model inputs, we calculated the parameter estimates and posterior probabilities of the full model (i.e., the one containing coupling parameters from dlPFC-BA46 to vmPFC and vice versa for all choice types and inter-trial fixation, as shown in Figure 3). Parameter estimates were computed using Bayesian parameter averaging (BPA) over subjects (Kasess et al., 2010). For completeness, we also tested the effective connectivity parameters from dlPFC-BA46 to vmPFC using two-tailed, one-sample *t*-tests against zero across individuals.

### Prediction exercise

We tested the hypothesis that the effective connectivity from left dlPFC-BA46 to vmPFC predicts between-subjects differences in the discount rate using the following out-of-sample prediction exercise. For every subject, we estimated the following linear regression using elastic net regularization (Zou and Hastie, 2005) (alpha parameter = 0.3) on the other N−1 subjects.

$$y=\beta_{0}+x_{1}\beta_{1}+x_{2}\beta_{2}+\cdots +x_{p}\beta_{p}$$

Where the dependent variable y was the log of the individual discount rates (*k*) and the explanatory variables x_1_, …, x_*p*_ were the complete set of estimated DCM parameters for each functional run (exclusive of the hemodynamic parameters) listed in Table 7. One advantage of the elastic net regularization is that the regression model is more robust to correlated predictor variables such as the DCM parameters for separate runs from the same subject.

We then used the fitted coefficients from the elastic net model to predict if the discount parameter for the excluded individual was above or below the mean value for the N−1 subjects. The procedure was repeated for every subject. Finally, we computed the balanced accuracy of the prediction using the confusion matrix, in which the rows represent the true labels and the columns represent the predicted labels, generated by our classification results (Brodersen et al., 2010). Briefly, this method controls for any imbalance in the data classes that may bias the classifier accuracy. The balanced accuracy is computed as

$$\frac{1}{2}\left(\frac{\text{TP}}{\text{TP}+\text{FN}}+\frac{\text{TN}}{\text{TN}+\text{FP}}\right)\text{​},$$

where TP, FN, TN, and FP represent the number of true positives, false negatives, true negatives, and false positives respectively.

In order to further test the specificity of our findings, we carried out different versions of this prediction exercise, in which subsets of the DCM parameters were excluded (see the Results section for details), or other candidate regions replaced left dlPFC-BA46 in the DCM. In all cases, we used the same fully connected DCM model with a fixed input to dlPFC-BA46 (or its replacement, when appropriate) on accepted trials and an input parametrically varying with the subjective value of the delayed reward to vmPFC.

### Specificity tests

These tests were designed to test the specificity of left BA46 activity and connectivity on the results. To do this, we repeated the DCM and prediction exercises described above using the left dlPFC-BA9 region listed in Table 6, or an ROI created by mirroring the 10 mm sphere centered on the estimated coordinates from Figner et al. (2010) to the right hemisphere.

## Results

### Choice behavior

We began the analysis by estimating individual discount rates (denoted by *k*) using maximum likelihood, and under the well-validated assumption that subjects exhibit hyperbolic discounting (Frederick et al., 2002; Green and Myerson, 2004; McKerchar et al., 2009). These estimates also allowed us to compute the discounted stimulus value (*dSV*) that each subject assigned to each option. As shown in Figure 1B, which depicts the group's psychometric choice curve, the estimated values provided a good description of the choice data. Figure 1C provides an ordered histogram of the individual estimates of the discount *parameter k* (with larger values denoting more frequent choices for immediate reward), and shows that there were sizable differences across individuals. This is important because one of the goals of the study is to relate individual differences in brain activity to differences in discounting behavior.

### Reaction times

Subjects responded well under the time limit of 3 s for both immediate [mean = 1.22 s, *SD* = 0.24 s, *t*_(26)_ = −39.2, *p* < 0.001] and delayed choices [mean = 1.20 s, *SD* = 0.25 s, *t*_(26)_ = −37.2, *p* < 0.001]. An ANOVA on reaction times as a function of choice (accept delayed offer vs. take money now) and group (those who chose to wait for delayed rewards on the majority of trials—wait group, WG—vs. those who most often took the money available today—now group, NG) showed no main effects of choice or group [*F*_(1, 48)_ = 0.10, *p* = 0.76 and *F*_(1, 48)_ = 0.49, *p* = 0.49, respectively]. However, there was an interaction between the tendency to wait and choice [*F*_(1, 48)_ = 4.79, *p* < 0.05]. This interaction was driven by the fact that WG subjects showed faster reaction times when choosing the delayed reward (mean = 1133 ± 70 ms) than when choosing the immediate reward [mean = 1277 ± 63 ms; *t*_(14)_ = −3.99, *p* < 0.001], whereas NG subjects had slower reaction times when choosing the delayed reward (mean = 1278 ± 62 ms) compared to immediate rewards [mean = 1143 ± 60 ms; *t*_(11)_ = 2.58, *p* < 0.05].

### GLM analyses localizing the dlPFC and vmPFC ROIs

A central goal of this study was to investigate the role of effective connectivity between the area of vmPFC that has been widely associated with the computation of stimulus values at the time of choice, and an area of left dlPFC, in BA 46, that has been shown to exert a causal influence on discounting behavior in monetary intertemporal choices and implicated in self-control processes in various domains. (See the Introduction and Discussion for more details). In order to carry out the connectivity analyses, we first needed to localize these two brain regions in our sample.

To do this, we first estimated a GLM of the BOLD responses that contained *dSV* and *Accept* (defined as 1 if the subject chose to accept the delayed option, and 0 otherwise) as parametric modulators at the time of decision (GLM-dSV). Based on previous studies, we expected that activity in vmPFC, PCC, and vStr would correlate with the *dSV* regressor, as these areas have been shown consistently to encode subjective values at the time of choice (Bartra et al., 2013; Clithero and Rangel, 2013). Note that in our task the immediate option was invariant ($25), whereas the delayed option changed every trial. Therefore, all trial-wise variation in the value of the delayed option is captured by *dSV*, even if the brain computes relative value signals (e.g., *dSV* − 25 or 25 - *dSV*).

The contrast for the *Accept* regressor showed that, after controlling for *dSV*, a region of left dlPFC in BA 46 was more active when subjects chose the larger, delayed option (*p* < 0.05, SVC; Table 2). No regions were more active when declining the larger delayed reward in favor of the $25 today. Note that this increased activity when accepting delayed options is present *after* controlling for *dSV*, indicating that it does not reflect a mere tendency of the subjects to choose larger rewards more frequently (indeed 12 out of 27 subjects in our sample choose the objectively smaller reward today most often).

**Table 2:**
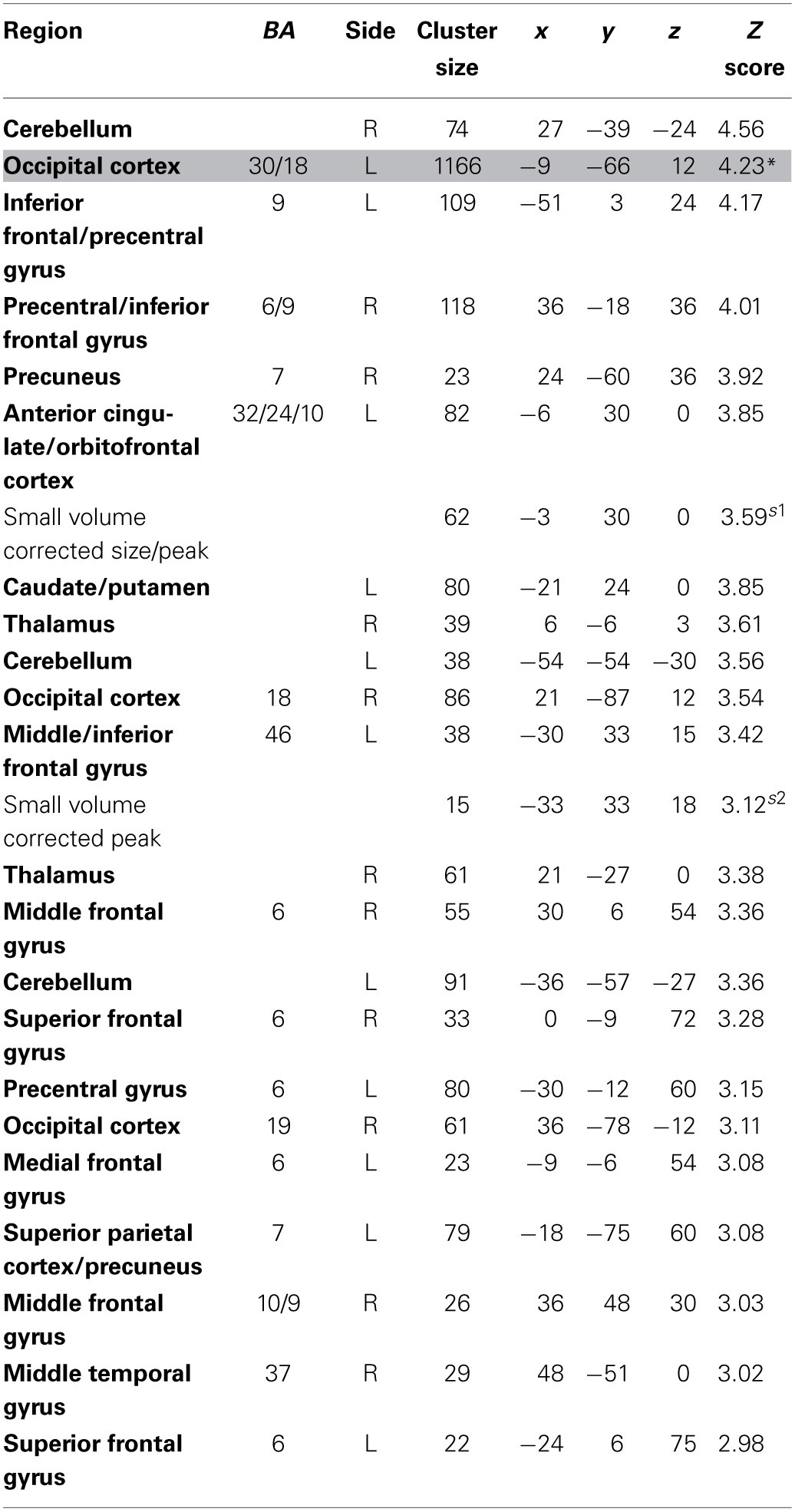
**Regions more active when accepting delayed rewards controlling for discounted stimulus value in GLM-dSV**.

*BA, Brodmann's Area*.

*Height threshold t = 2.78 (p < 0.005) and extent of 20 voxels for table inclusion*.

Gray highlighting and ^*^ signify that the activation survives whole brain correction (p < 0.05) for multiple comparisons at the cluster level.

*^s1^Signifies that the activation survives small volume correction within an anatomical mask of vmPFC*.

*^s2^Signifies that the activation survives small volume correction within a 10 mm sphere centered on the estimated MNI coordinates from Figner et al. (2010) (x, y, z = −36, 30, 27)*.

*Peak voxel coordinates and cluster sizes within the small volume correction masks are listed in plain text below the corresponding clusters identified in the whole brain analysis*.

The contrast for *dSV* revealed a large cluster (1277 voxels) with a peak in the middle frontal gyrus (Brodmann Area 6), extending into the putamen that was positively correlated with *dSV* (*p* < 0.05 WBC; Table 3), but, to our surprise, no significant activity in the vmPFC region consistently linked to value computation at the group level (Bartra et al., 2013; Clithero and Rangel, 2013). In addition, none of the regions from the meta-analysis of negative correlations with subjective value in Bartra et al. (2013) showed decreasing activity as a function of *dSV* at whole brain or small volume corrected thresholds. Although there was no significant activity in canonical reward regions at the group level, inspection of individual participant results revealed positive correlations with *dSV* within the vmPFC for many individuals. However, there was also a large fraction of participants who showed negative correlations with *dSV* in vmPFC, resulting in a summation of signed *t*-test coefficients that was close to zero at the group level.

**Table 3:**
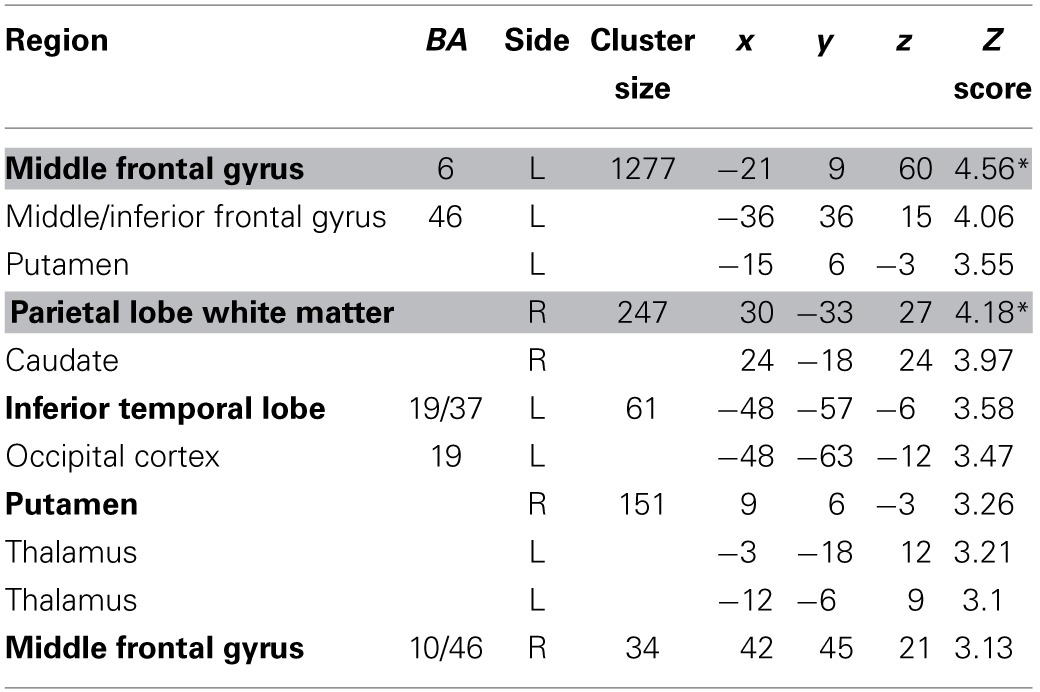
**Regions reflecting discounted stimulus value at the time of choice in GLM-dSV**.

*BA, Brodmann's Area*.

*Height threshold t = 2.78 (p < 0.005) and extent of 20 voxels for table inclusion*.

Gray highlighting and ^*^ signify that the activation survives whole brain correction (p < 0.05) for multiple comparisons at the cluster level.

*Labels in bold text are for the peak voxel within each cluster. Labels in plain text identify local maxima more than 8 mm apart in different anatomical regions of larger clusters*.

**Figure 2:**
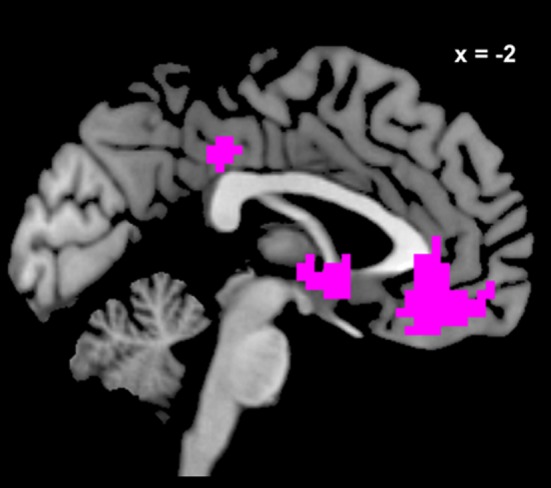
**Voxels in meta-analytically defined reward value regions whose pattern of activity is better explained by a GLM including *dSV* as a regressor than a reduced version of the GLM omitting *dSV*.** Voxels in violet are those within a mask of reward value sensitive regions including vmPFC, vStr, and PCC created from recent meta-analyses on reward value computation (Bartra et al., 2013; Clithero and Rangel, 2013) where the exceedance probability for GLM-dSV compared to the reduced version without *dSV* is 0.90 or higher. The exceedance probability was 0.90 or higher for the version of GLM-dSV including *dSV* in 83% of voxels within the meta-analysis conjunction.

In order to examine the association between *dSV* and BOLD signals at the group level in a manner independent of the sign (+/−) of this relationship, we re-estimated GLM-dSV, as well as a reduced version of this model excluding the *dSV* regressor, using Variational Bayes (see Materials and Methods). The logic of comparing the original version of GLM-dSV with the reduced model excluding *dSV* is that any differences in their fits to the data can be attributed to variance explained by *dSV*. Critically for our purposes, the amount of variance explained is unchanged by the sign of the regression coefficient allowing us to compute random effects statistics across individuals showing positive and negative effects of *dSV*. Following estimation of these models at the individual subject level, we compared their relative probabilities given the data from all subjects using BMS within a mask of reward value sensitive regions including vmPFC, vStr, and PCC created from recent meta-analyses on reward value computation (Bartra et al., 2013; Clithero and Rangel, 2013). Here we compared the models based on their respective exceedance probabilities, which are measures that corresponds to the belief that a particular model is more likely than any other in the test set given the data from all participants. Figure 2 shows all voxels in this mask where the exceedance probability for GLM-dSV compared to the reduced version without *dSV* is 0.90 or higher. Quantitatively, 83% of voxels in the meta-analysis conjunction had an exceedance probability of 0.90 or higher for the version of GLM-dSV including *dSV*, whereas only 4% had a value of 0.90 or higher for reduced GLM-dSV. This indicates that the *dSV* regressor explains a significant amount of the variance in the BOLD response in vmPFC, vStr, and PCC.

Given that previous studies have found evidence consistent with the encoding of relative value signals in vmPFC at the time of choice (Boorman et al., 2009; Lim et al., 2011; Hunt et al., 2012), we carried out an additional generalized linear model (GLM-rdSV). We hypothesized that there might be individual differences in the computation of the relative subjective value. In fact, a class of popular models in behavioral economics predicts that subjects will use as their reference item (i.e., the one that is subtracted when computing relative value) the option that they choose most frequently (Koszegi and Rabin, 2006). Based on this, we defined a relative discounted subjective value regressor (*rdSV*) that is given by *dSV* − 25 for those that picked the delayed option more than 50% of the time, and by 25 − *dSV* for those that select the immediate option most frequently.

**Figure 3:**
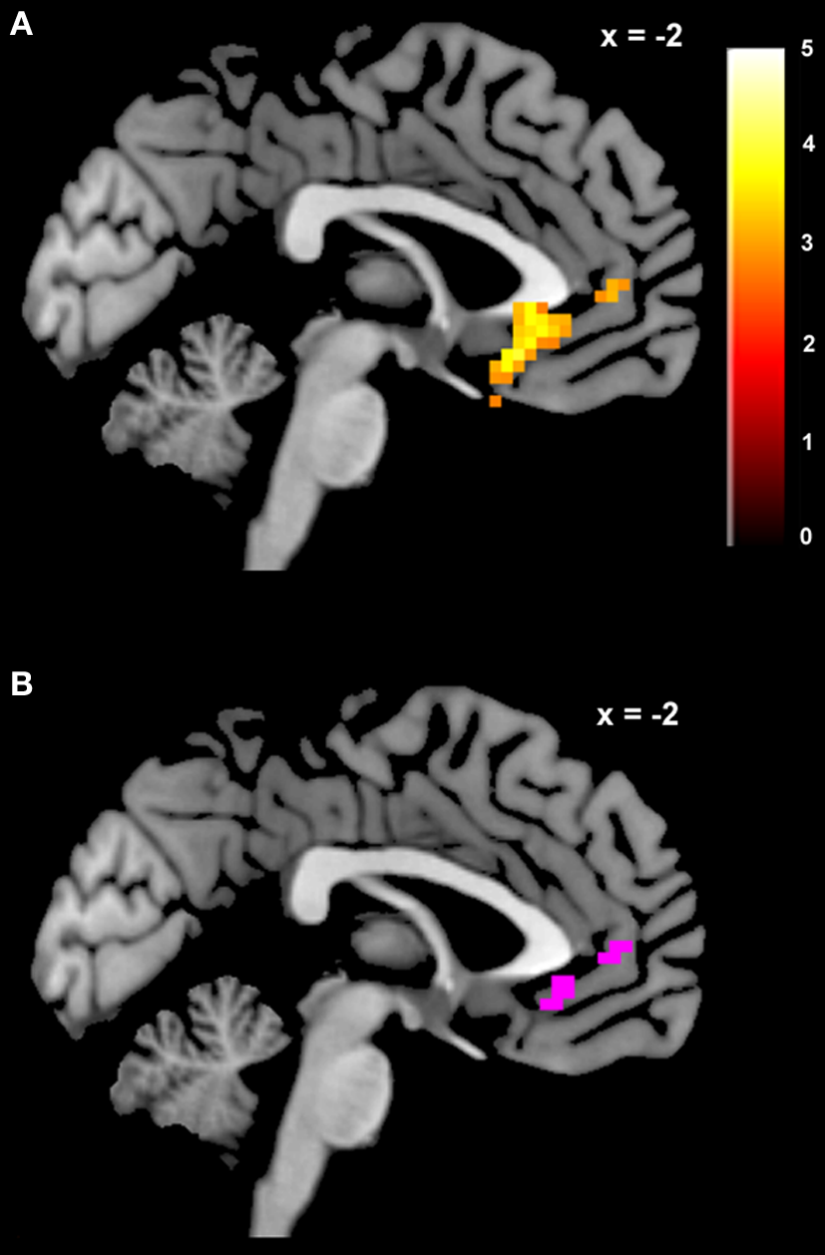
**Areas correlated with the *rdSV* regressor from GLM-rdSV**. **(A)** A region of vmPFC showing increased activity as a function of *rdSV* (*p* < 0.05 SVC). **(B)** Voxels in vmPFC where the activation for *rdSV* in the current study overlaps with significant voxels in meta-analyses of positive correlations with subjective value by Bartra et al. (2013) and Clithero and Rangel (2013). All voxels shown in violet are significant in all three studies.

**Figure 4:**
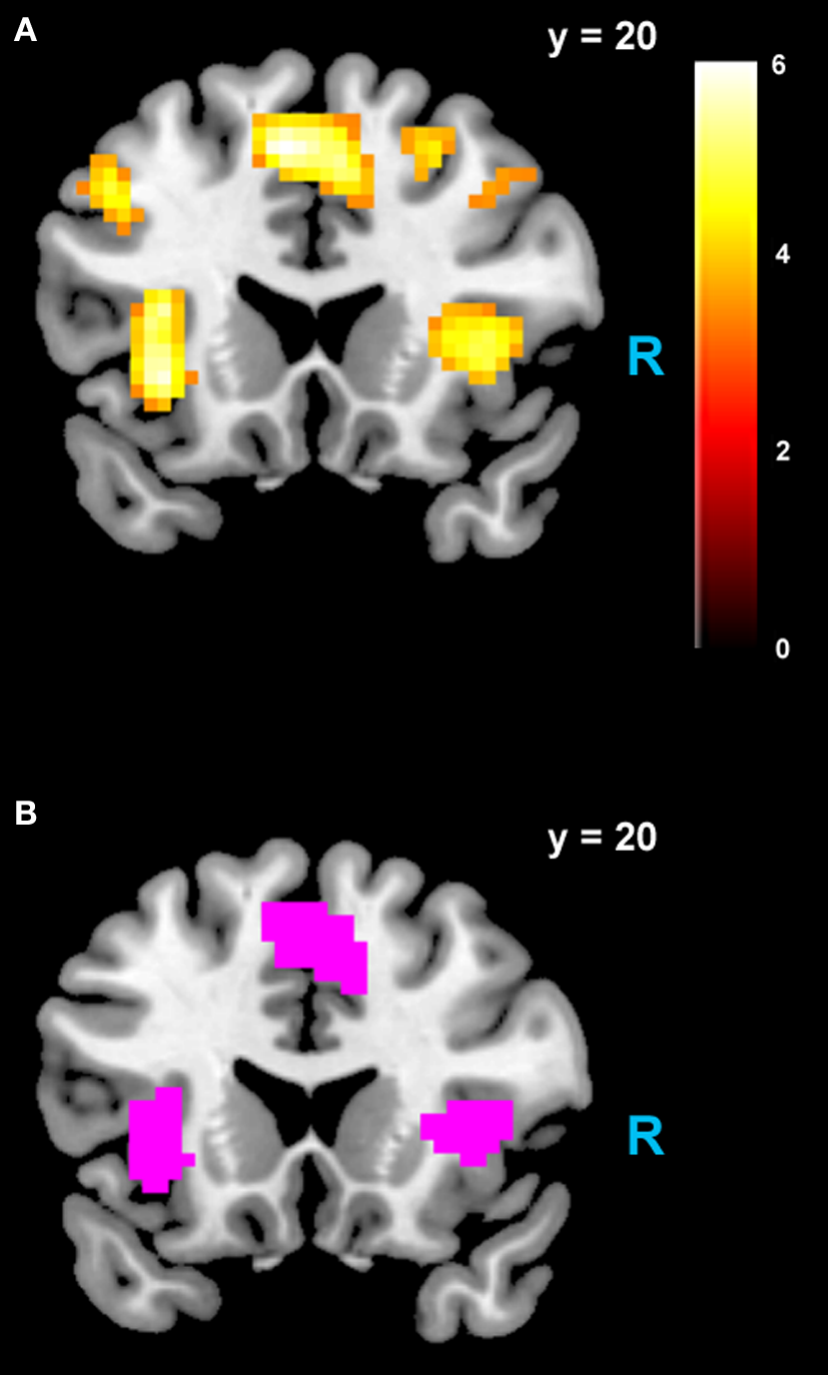
**Areas negatively correlated with the *rdSV* regressor from GLM-rdSV**. **(A)** Regions of the dmPFC and AI where activity decreased as a function of *rdSV* (*p* < 0.05 WBC). **(B)** Voxels in dmPFC and AI where responses to *rdSV* overlap with the meta-analyses results for regions that negatively correlated with subjective value at the time of choice in Bartra et al. (2013). All voxels shown in violet are significant in both studies.

We estimated a new GLM (GLM-rdSV) with *rdSV* and *Accept* as parametric modulators at the time of decision. Consistent with the *post-hoc* hypothesis that value computations were made relative to the most frequent choice, we found that BOLD responses in vmPFC (*p* < 0.05 SVC, Figure 3A; Table 4) and the anterior superior temporal gyri (*p* < 0.05 WBC) were positively correlated with the modified value regressor. In addition, several regions including the anterior insula (AI), dorsomedial prefrontal cortex (dmPFC), inferior parietal cortex, middle frontal gyri, and posterior cingulate showed negative correlations with *rdSV* (*p* < 0.05 WBC; Figure 4A; Table 5). Both the vmPFC region that positively correlated with *rdSV* and the portions of the AI and dmPFC that negatively correlated with *rdSV* show considerable overlap with results from recent meta-analyses (Bartra et al., 2013; Clithero and Rangel, 2013) of reward value representation (Figures 3B, 4B). This overlap with previous results for both positive and negative correlations with subjective value suggests that there is significant variation between subjects in how discounted subjective values are computed and that this computation may be related to choice frequencies (e.g., most often wait or rarely wait) consistent with our *post-hoc* hypothesis. We do not presume or infer any causal relationships between choice frequency, and the directionality of relative discounted subjective value computations from these results, and it may be that a third as yet unknown variable drives choice preference, value computation, or both. However, this analysis provides us with a sample-specific ROI for vmPFC in which to test our main hypothesis about dlPFC modulation and the prediction of individual differences.

**Table 4:**
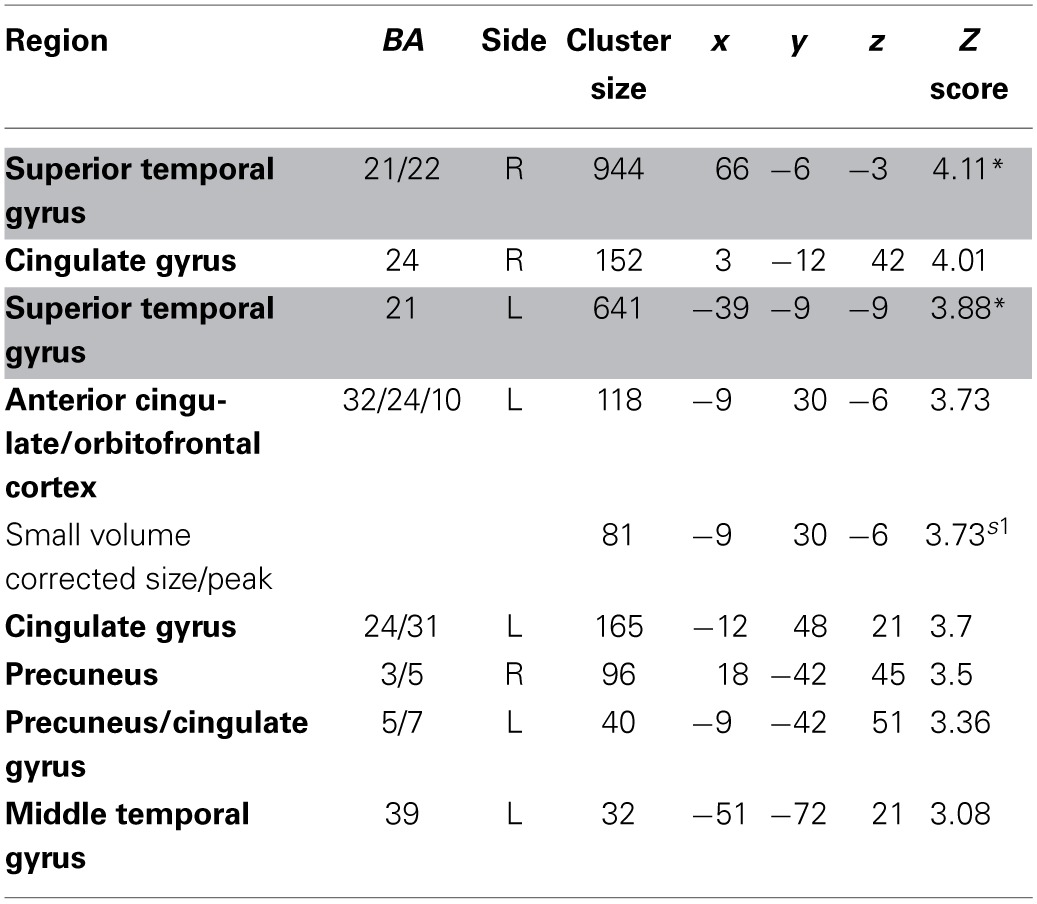
**Regions positively correlated with relative discounted stimulus value at the time of choice in GLM-rdSV**.

*BA, Brodmann's Area*.

*Height threshold t = 2.78 (p < 0.005) and extent of 20 voxels for table inclusion*.

Gray highlighting and ^*^ signify that the activation survives whole brain correction (p < 0.05) for multiple comparisons at the cluster level.

*^s1^Signifies that the activation survives small volume correction within an anatomical mask of vmPFC*.

*Peak voxel coordinates and cluster sizes within the small volume correction masks are listed in plain text below the corresponding clusters identified in the whole brain analysis*.

**Table 5:**
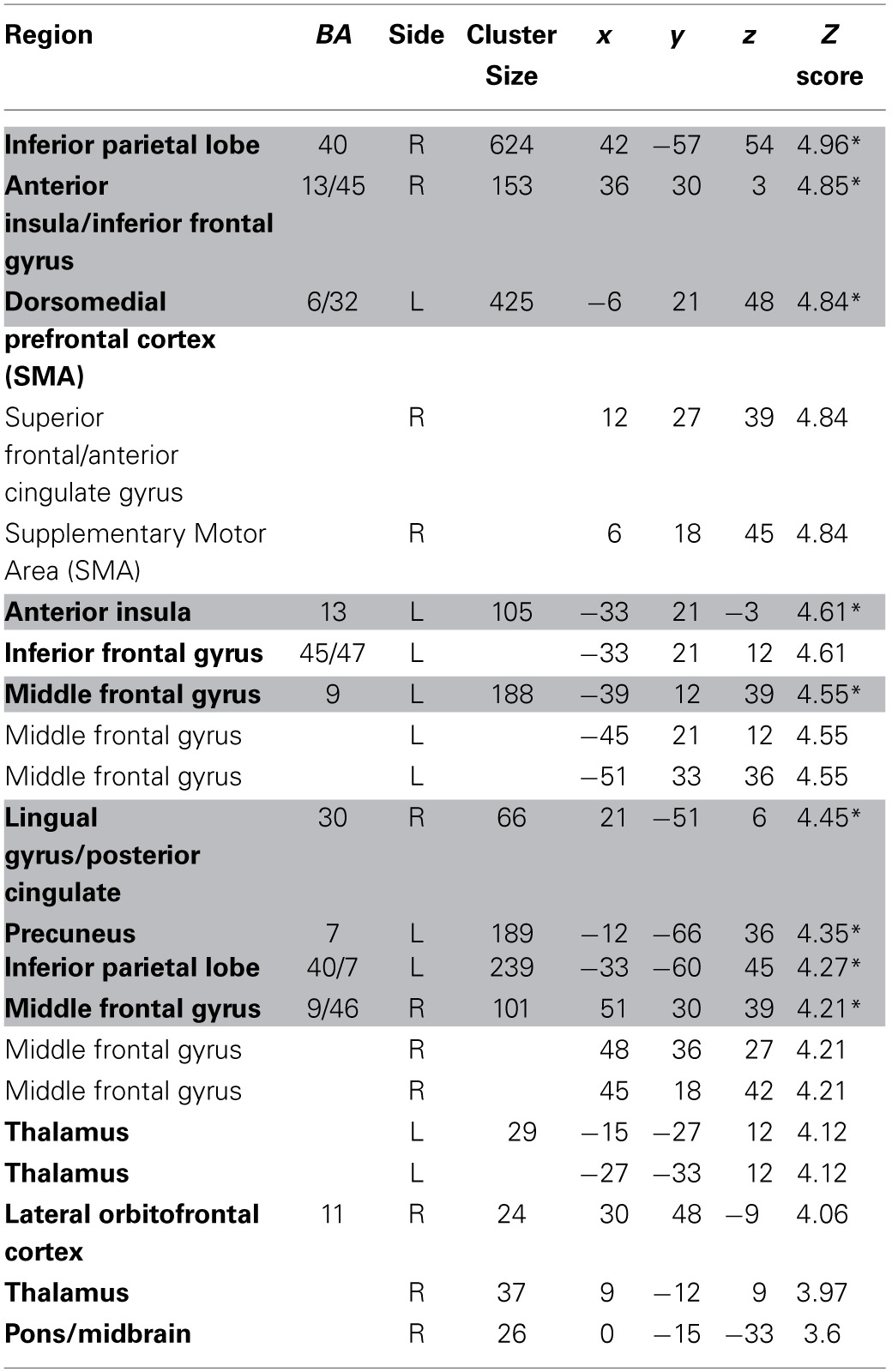
**Regions negatively correlated with relative discounted stimulus value at the time of choice in GLM-rdSV**.

*BA, Brodmann's Area*.

*Height threshold t = 3.44 (p < 0.001) and extent of 20 voxels for table inclusion. A larger individual voxel threshold was used here to separate large clusters*.

Gray highlighting and ^*^ signify that the activation survives whole brain correction (p < 0.05) for multiple comparisons at the cluster level.

*Labels in bold text are for the peak voxel within each cluster. Labels in plain text identify local maxima more than 8 mm apart in different anatomical regions of larger clusters*.

**Figure 5:**
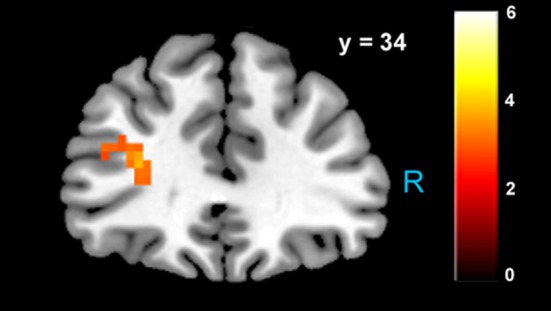
**Increased activity in left dlPFC when choosing to accept larger, delayed rewards after controlling for subjective value (*p* < 0.05, SVC)**. The region of BA 46 shown here lies directly beneath the TMS stimulation site from Figner et al. (2010) that showed causal effects on temporal discounting behavior.

Consistent with GLM-dSV, GLM-rdSV showed that regions of left dlPFC in BA 46 and 9 were more active when subjects chose the larger, delayed option (*p* < 0.05, SVC; Figure 5; Table 6). Just as in GLM-dSV, no regions were more active when declining the larger delayed reward in favor of the $25 today. Note that both GLM-dSV and GLM-rdSV control for the value of delayed rewards in a similar manner. The variance explained is the same in both models because in the individual subject GLMs only the sign of *dSV* regressor, and therefore, the sign on the regression coefficients changes while the explanatory power of the regressor remains the same.

**Table 6:**
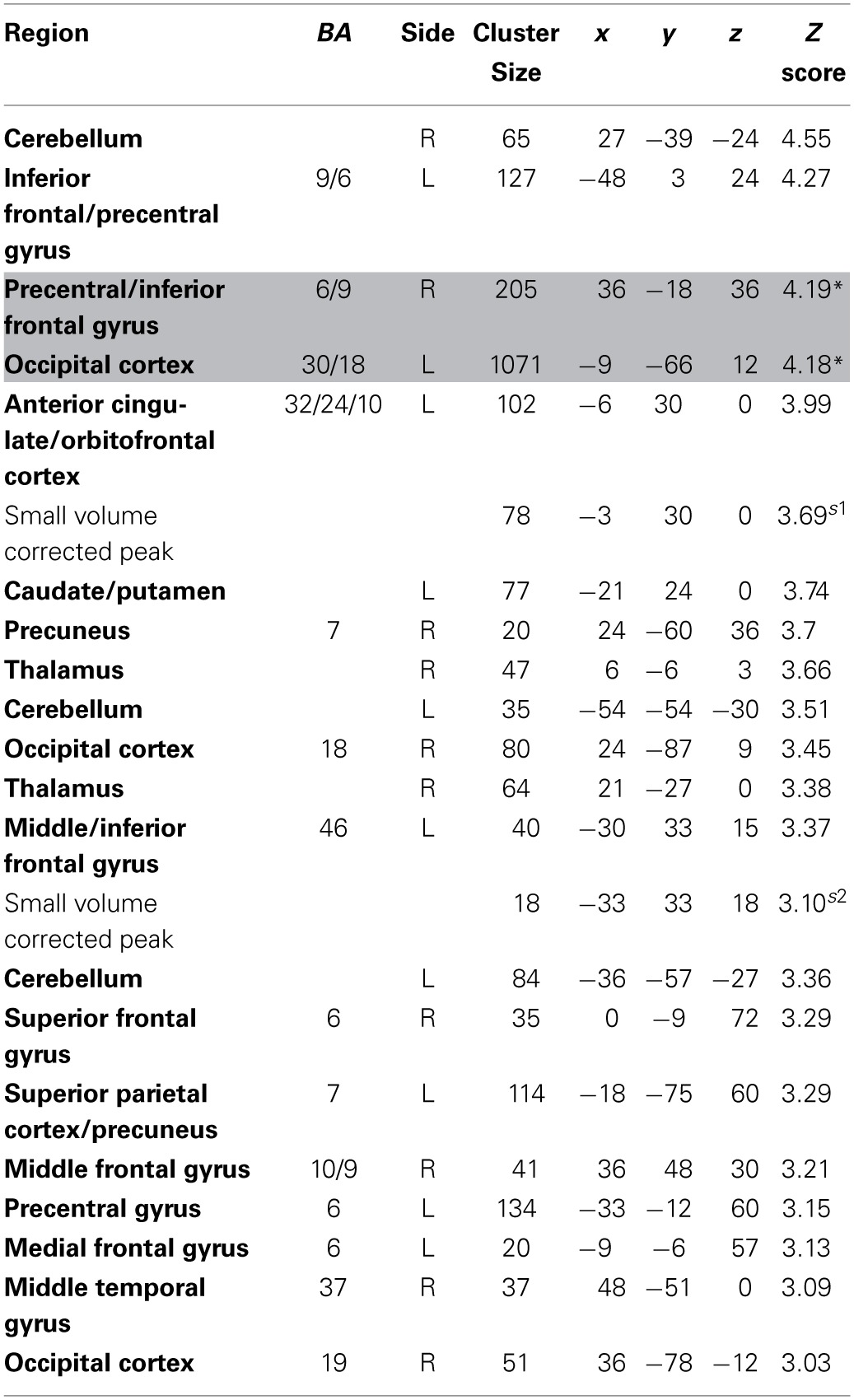
**Regions more active when accepting delayed rewards controlling for discounted stimulus value in GLM-rdSV**.

*BA, Brodmann's Area*.

Height threshold t = 2.78 (p < 0.005) and extent of 20 voxels for table inclusion

Gray highlighting and ^*^signify that the activation survives whole brain correction (p < 0.05) for multiple comparisons at the cluster level.

*^s1^Signifies that the activation survives small volume correction within an anatomical mask of vmPFC*.

*^s2^Signifies that the activation survives small volume correction within a 10 mm sphere centered on the estimated MNI coordinates from Figner et al. (2010) (x, y, z = −36, 30, 27)*.

*Peak voxel coordinates and cluster sizes within the small volume correction masks are listed in plain text below the corresponding clusters identified in the whole brain analysis*.

### Tests of effective connectivity

Next, we used the ROIs in dlPFC-BA46 and vmPFC to test our first hypothesis; namely, that effective connectivity from left dlPFC-BA46 to vmPFC plays a critical role in delaying gratification. This test was carried out on time courses extracted from the vmPFC and dlPFC-BA46 ROIs identified in GLM-rdSV. We focused on the ROI in BA46 because a previous TMS study found a causal role for this region in choosing to wait for larger delayed rewards in monetary intertemporal choices (Figner et al., 2010). Furthermore, our previous effective connectivity analyses of dietary self-control choices suggested other dlPFC regions active during self-control choices (e.g., BA9) might work through BA46 to modulate vmPFC (Hare et al., 2009). As explained in the Materials and Methods section, the test was performed in several steps.

**Figure 6:**
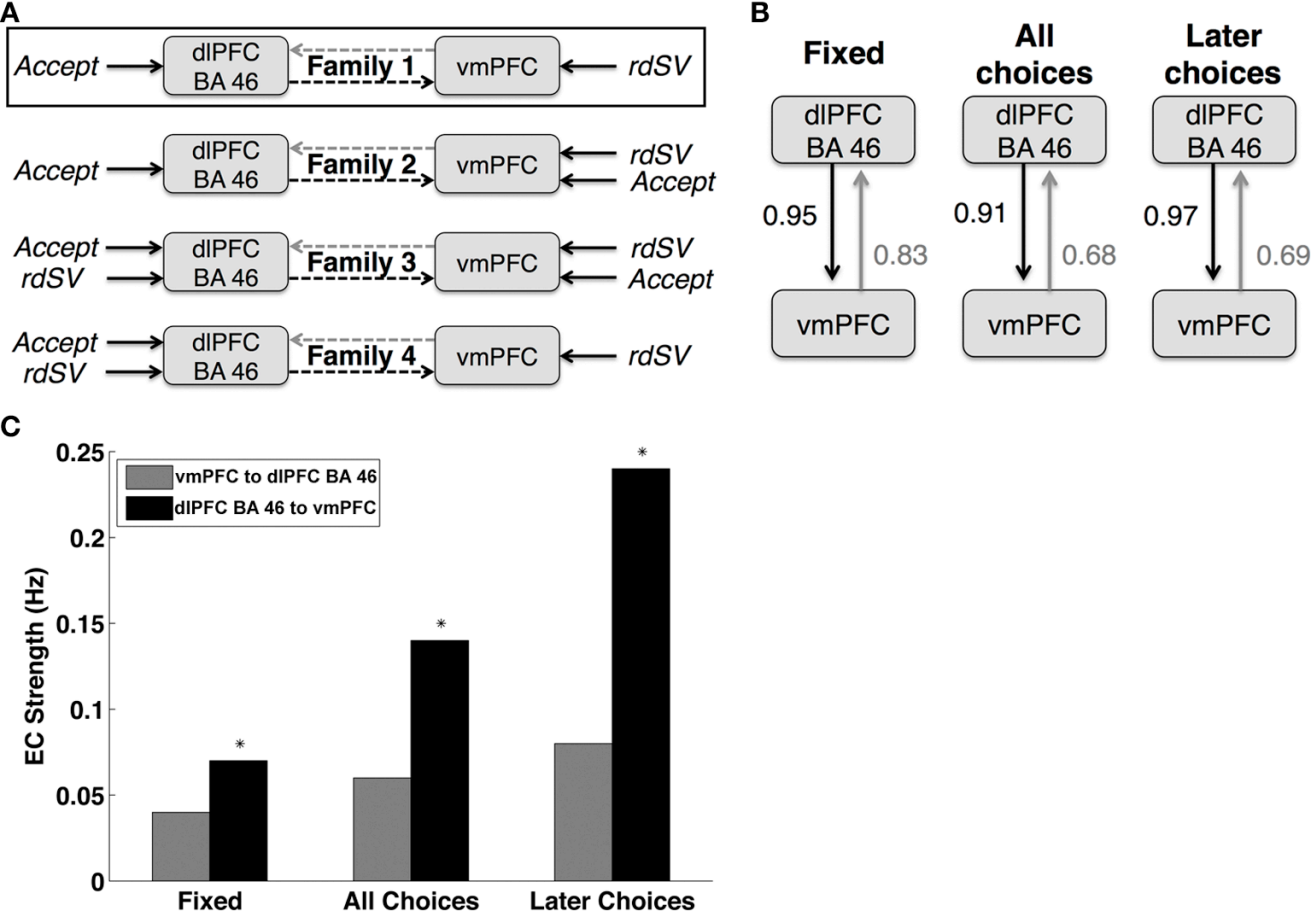
**Dynamic causal modeling results**. **(A)** Schematic representations of the four DCM families compared in order to optimize the task related driving input to dlPFC BA 46 and vmPFC. Bayesian Model comparison showed that Family 1, outlined in black, was the most likely description of the data generating process. **(B)** Diagram of the fully connected model from the most likely family showing the posterior probabilities of coupling or coupling modulation greater than zero between vmPFC to dlPFC BA 46. Fixed refers to the baseline coupling during all time points. All Choices refers coupling modulation at the time of decision for all choices regardless of whether the immediate or delayed option was selected. Later Choices refers to coupling modulation during only those decisions when the larger, delayed option was chosen. **(C)** Bar chart showing the effective connectivity (EC) strengths in Hertz (Hz) between dlPFC BA 46 and vmPFC at different task periods. The colors and labels correspond to the diagram in panel **(B)**. Asterisks indicate DCM parameters that are significantly different from zero when tested using both Bayesian parameter averaging (posterior probability > 0.90) and one sample *t*-tests (*p* < 0.01).

First, we estimated 4 different DCM families that were grouped based on how the experimental variables *rdSV* and *Accept* entered into the model as driving inputs (Figure 6A). Each family contained 16 models that varied on how the vmPFC and dlPFC-BA46 affect each other as a function of three task events: fixation, choice periods, and choice periods when the delayed option is selected.

Next, we used BMS to identify the most likely family of models. We compared the models based on their respective exceedance probabilities, a measure of whether particular model is more likely than any other in the test set given the data from all participants. The most likely model family (exceedance probability = 0.87), shown in Figure 6A, had two driving inputs (i.e., direct influences): an input to vmPFC given by the *rdSV* of the delayed option on every trial, and an input to dlPFC-BA46 given by *Accept*.

Lastly, we examined the effective connectivity parameters between dlPFC-BA46 and vmPFC using BPA on the fully connected model (Figure 6B) with the most likely experimental inputs across subjects. We found increased signaling from dlPFC-BA46 to vmPFC at the time of choice relative to inter-trial fixation times, and further increases when subjects selected the later option (posterior probability > 0.90 and 0.95, respectively; Figure 6C). In contrast, the signaling in the other direction was not significantly different from zero. For completeness, we also compared the estimated DCM coefficients using one-sample *t*-tests, which lead to the same conclusion: effective connectivity parameters from dlPFC-BA46 to vmPFC increased during all choices and further increased when subjects selected the later option [*t*_(26)_ = 2.65 and *t*_(26)_ = 3.80 respectively; *p* < 0.01], but signaling in the opposite direction did not increase significantly during any task events, suggesting that there is increased connectivity from dlPFC-BA46 to vmPFC during decisions to wait for larger delayed rewards, but not in the other direction.

### Between-subjects prediction

Next, we used the results of the DCM, to test our second hypothesis; namely, that it is possible to use inter-individual differences in the strength of effective connectivity between dlPFC-BA46 and vmPFC, as well as differences in local responses in those regions, to predict differences in discount rates.

For each subject we estimated an elastic net regression model using only the data from the N−1 other subjects, with discount rates as the dependent variable, and the estimated DCM parameters as the predictors. The estimated parameters of the model were then used to predict whether the discount rate of the excluded subject was above or below the mean of the group. The procedure was repeated to obtain a prediction for each subject. We found that the mean balanced accuracy (MBA) across all subjects was 71% correct (95% posterior probability interval = 54 − 85%). In a complimentary analysis, we tested how well the continuous discount rate rankings (e.g., 1 = highest to 27 = lowest) estimated from the DCM parameters matched to those estimated from behavior, and found a significant correlation (Spearman's rho = 0.42, *p* < 0.02). Thus, the elastic net regression model can accurately predict both binary high low labels and the continuous ordinal rank of discount rates using DCM parameters.

Next, we compared the accuracy of several versions of this prediction exercise, to test the specific role of the various components of the DCM in predicting the individual discount rates. Note that all parameters were estimated in the fully connected version of the model (shown in Figure 6) and were simply omitted from the elastic net regressions during these tests. The logic of these tests is as follows: the prediction accuracy of a regression model that excludes a key parameter should drop, whereas excluding a parameter that does not play a role in intertemporal choice should not affect the model's ability to predict the discount rates. The first test excluded the local driving input response parameters in vmPFC (Spearman's rho = 0.14, *p* = 0.48; MBA = 65%; 95% post. prob. int. = 47 − 80%). The second test excluded the local driving input response parameters for dlPFC-BA46 (Spearman's rho = −0.03, *p* = 0.90; MBA = 60%; 95% post. prob. int. = 44 − 76%). The third test excluded the effective connectivity parameters from dlPFC-BA46 to vmPFC (Spearman's rho = −0.12, *p* = 0.54; MBA = 58%; 95% post. prob. int. = 41 − 74%). The fourth test excluded the effective connectivity parameters in the other direction (Spearman's rho = 0.02, *p* = 0.91; MBA = 54%; 95% post. prob. int. = 37 − 70%).

We found that omitting effective connectivity parameters between dlPFC-BA46 and vmPFC in either direction, or parameters measuring local task induced responses within dlPFC-BA46 or vmPFC reduced the accuracy to chance levels. Together, these findings show that the local responses in both areas, as well as both directions of effective connectivity between dlPFC and vmPFC, are critical for explaining the individual differences in discounting. Table 7 lists the relative size and direction of the effects of each DCM parameter on discount rates when estimating the model over all 27 participants.

**Table 7:** **Regression coefficients predicting log(k) as a function of DCM parameters**.

**Task period**	**DCM parameter**	**Elastic net parameter**
Fixed	v −> d	0.6
	d −> v	−1.9
All choices	v −> d	−0.7
	v self	−0.1
	d −> v	3.3
	d self	−1.6
Later choices	v −> d	0.8
	v self	0.0
	d −> v	−0.9
	d self	0.2
Driving inputs	Value −> v	0.4
	Accept −> d	0.6

*This table reports the regression coefficients from the elastic net regression using the DCM parameters specified above to predict discount rates (log(k))*.

*The regression is identical to those used in the prediction exercises described in the main text except that it was run with all 27 subjects at once. The regressions were estimated using the DCM parameters for both functional MRI runs separately and the values listed in this table represent the average of coefficients across runs for conciseness and clarity*.

*Note that the DCM parameters have been z-scored across subjects so that comparing the coefficients across parameters shows the relative size and direction of the effects of each DCM parameter on discount rates. See the main text of the Results section for quantification of the influence of each parameter on the model's ability to predict discount rates. Also, recall that smaller k values indicate lower discount rates and, therefore, parameters with negative regression coefficients increase the likelihood of choosing delayed reward options in our temporal discounting paradigm*.

*The labels Fixed, All Choices, Later Choices, and Driving Inputs correspond the portions of the DCM described in the main text and shown in Figure 5*.

*The label v −> d refers to signaling from vmPFC to dlPFC-BA 46*.

*The label d −> v refers to signaling from dlPFC-BA 46 to vmPFC*.

*The label v or d self refers to the parameters for the inhibitory self-connections to each region at the specified time points*.

*The label Value −> v refers to an input equal to rdSV from GLM-rdSV into vmPFC*.

*The label Accept −> d refers to an input equal the Accept regressor specifying later choices in GLM-rdSV*.

Finally, we tested the specificity of these results with regard to the left dlPFC BA46 region. We replaced left dlPFC BA46 in DCMs using the vmPFC with either the more posterior left dlPFC BA9 ROI that was also found to increase its activity when subjects chose the delayed rewards, or with an ROI created by mirroring the 10 mm sphere centered on the estimated coordinates from Figner et al. (2010) to the right hemisphere. This resulted in two new DCMs and elastic net regression models. None of these combinations yielded significantly better than chance predictions (best MBA = 55%) or significant correlations with the true discount rates. The results replacing left dlPFC-BA 46 with the analogous region in the right hemisphere are consistent with previous TMS results showing that only stimulation of the left hemisphere impacted choices for the delayed monetary rewards (Figner et al., 2010).

## Discussion

The results in this paper, in conjunction with previous reports (Hare et al., 2009, 2011a; Harris et al., 2013), suggest that a similar set of computational and neurobiological mechanisms are at work in tasks involving the delay of gratification in dietary and monetary intertemporal choices. In particular, we found that left dlPFC BA46 becomes more active in trials in which subjects choose the delayed option, which on average requires more self-control. We also found that the connectivity from left dlPFC BA-46 to a region of vmPFC widely associated with the computation of stimulus values (Bartra et al., 2013; Clithero and Rangel, 2013), increased at the time of choice, and especially during trials in which subjects chose to wait for the delayed reward. In addition, we were able to explain between-subject differences in discount rates using the estimated parameters from a DCM including the activity within dlPFC BA-46 and vmPFC, and the coupling between them, but only if the effective connectivity parameters between the two areas were included.

These results parallel previous findings in the domain of dietary choice, in which individuals chose among foods that differed in their tastiness and healthiness (Hare et al., 2009, 2011a; Harris et al., 2013). Although an explicit between-subject prediction exercise was not performed in those previous studies, the data indicate a central role for dlPFC-vmPFC interactions in dietary self-control. This suggests that the mechanisms mediating self-control described in the Introduction are at work in both tasks, and thus helps to advance our understanding of common computational and neurobiological components of various forms of self-control. In this model, vmPFC computes the value of options by identifying its various attributes, assigning value to them, and then integrating them into a net value for the option. A critical component of the model is that basic attributes (like immediate monetary payoffs, or the tastiness of foods) are preferentially incorporated into the values computed in vmPFC, but that more abstract attributes (like delayed monetary payoffs, or the healthiness of foods) are generally given less weight unless left dlPFC comes online and modulates activity in vmPFC, so that it weights all attributes according to the current goals (e.g., eat healthy or maximize monetary payoff). Note that the types of attributes that need to be represented and evaluated in both types of tasks are different, but that poor self-control could be attributed to the same source in both cases: reduced weighting of abstract attributes in vmPFC in the absence of dlPFC modulation.

One limitation of the study must be emphasized. Our experiment is not able to differentiate between heterogeneity in the discount rates attributable to patience or self-control abilities (potentially mediated by differences in dlPFC functioning or connectivity), and heterogeneity due to differences in individual circumstances (e.g., immediate budgetary constraints) that are not directly associated with patience or self-control. Differences in individual circumstances, therefore, do not enter our prediction model and may be one reason why the model is less than perfectly accurate. In other words, our analysis cannot indicate if less patient subjects failed to wait for delayed rewards because they are unable to do so, or because their best option was to take the immediate monetary payout.

These results provide novel interpretations of results in the sizable literature on intertemporal choice paradigms. Consider three important examples.

First, there has been a debate in the literature on whether or not there are multiple and competing value signals at work in self-control. In particular, previous findings (McClure et al., 2004, 2007) have been interpreted as suggesting that vmPFC-VStr and dlPFC compute parallel but distinct value signals, with a vmPFC-VStr valuation system placing more value on immediate, concrete outcomes, and areas such as dlPFC computing the value of long-term, abstract goals. In this view, the quality of decisions depends on competition between the two valuation systems. In contrast, others have proposed that one value system integrates information about all stimulus attributes, both immediate and long-term, to form an overall value for the stimulus (Kable and Glimcher, 2007, 2010). In this view, the quality of decision-making depends solely on the weighting of different stimulus attributes in value computation. The results here, and in previous work (Hare et al., 2009, 2011a), suggest an obvious way of reconciling both views. In this class of tasks, choices seem to be driven by the stimulus value signals encoded in a vmPFC-based valuation system, but the activation of dlPFC is critical for the deployment of self-control, because it appears to promote increased weighting of foresighted stimulus attributes in the vmPFC value signals as evidenced by increased effective connectivity to vmPFC during larger delayed choices.

Second, our results provide a mechanistic explanation of the influential study of Figner et al. (2010), which found that applying inhibitory TMS over left (but not right) dlPFC-BA46 resulted in a decrease in subjects' willingness to wait for delayed rewards (Figner et al., 2010). Consistent with the implication of a causal role for left dlPFC in self-control from these previous results, we find that this region is more active when subjects chose larger future rewards over payments on the same day, after controlling for the subjective value of the payments. Furthermore, our data and analyses indicate that the left BA46 region of dlPFC contributes to delaying gratification by influencing the valuation process in vmPFC at the time of choice, rather than intervening after valuation has occurred, as was previously suggested in Figner et al. (2010). The previous suggestion by Figner and colleagues was based on their finding that choices over delayed options, but not the attractiveness ratings of those delayed rewards were affected by TMS to left dlPFC. However, our data on effective connectivity from dlPFC to vmPFC at the time of choice are more consistent with a mechanism in which dlPFC activity directly impacts valuation processes at the time of choice. We note, however, that these results are not contrary to Figner and colleagues' assertion that the role of dlPFC is specific to decisions as opposed to outcome free ratings.

Third, recent EEG and fMRI studies have found that individual measurements of activity and connectivity within networks including left dlPFC taken at rest exhibited a sizable correlation with discount rates taken in separate behavioral tasks (Gianotti et al., 2012; Li et al., 2013). Similarly, a study of alcoholics found that responses in left dlPFC also correlated with behavior during intertemporal choices (Boettiger et al., 2007). Our results also provide a novel mechanistic explanation for these findings as a whole. Furthermore, our prediction exercises show that measures of effective connectivity between dlPFC and vmPFC are a critical aspect of being able to predict individual discount rates.

We investigated the specificity of the dlPFC-vmPFC interactions in self-control by repeating a similar exercise replacing left dlPFC-BA 46 with left dlPFC-BA 9 or right dlPFC-BA 46.

The specificity test using left dlPFC-BA 9 was motivated by the fact that this area was more active when subjects delayed gratification in previous dietary choice experiments (Hare et al., 2009, 2011a) as well the current monetary choice dataset, although the activity did not survive whole brain correction in the current sample. However, it did not result in significant correlations with or above average predictions of between-subject discount rates. This is consistent with our previous findings in dietary self-control where dlPFC-BA9 did not directly interact with vmPFC, but rather affected a more anterior region in BA46, near the region we find in the current intertemporal monetary choice task.

The intertemporal choice task utilized here, as well as the dietary choice task that we have used in our previous related work (Hare et al., 2009), examines the deployment of self-control in the context of goal-directed choice. Other types of self-regulation might be better characterized by competition between habitual and goal-directed systems (Dayan et al., 2006; Balleine et al., 2008; Rangel et al., 2008), or by the type of response inhibition associated with action control in paradigms such as the go/no-go, Flanker, or Stroop tasks (Wager et al., 2005; Congdon et al., 2010). A critical question for future work is to systematically investigate the commonalities and differences between these various sources of self-regulation.

Another avenue for further investigation is our finding that subjects appear to compute the discounted subjective value of delayed rewards relative to their most common choice, perhaps viewing this as a default. While not true in every case, the majority of subjects who most often chose the immediate reward appeared to positively encode a relative value signal in vmPFC equal to the difference between the immediate reward and the larger delayed reward (i.e., $25 − *dSV*). They also showed negative correlations with this relative value signal in a network of regions that includes dmPFC, AI, and parietal regions consistently shown to negatively correlate with SV (Bartra et al., 2013). This network has been implicated in computations related to conflict, error processing, decision difficulty, and evidence accumulation (Carter et al., 1998; Botvinick, 2007; Pochon et al., 2008; Venkatraman et al., 2009; Wunderlich et al., 2009; Hare et al., 2011b). On the other hand, subjects who most often waited for the delayed reward frequently encoded the opposite relative value signal of *dSV* − $25 in both sets of regions. This suggests that it is important to control for reference point variation across subjects when examining the neural correlates of subjective values at the group level, but further investigation of this issue is clearly warranted.

While our findings may at first seem contradictory to previous reports where all subjects showed positive correlations with a value signal proportional to *later reward—immediate reward*, this can potentially be explained by important methodological differences. Many previous studies of intertemporal choice have customized the offer sets for each participant to maintain an acceptance rate close to 50% for all subjects (Kable and Glimcher, 2007, 2010; Peters and Buchel, 2009, 2010). In contrast, we purposefully utilized the same offer set for all subjects to examine individual differences in neural responses. By keeping the response rate near 50% for all subjects, these previous studies may have also generated a more homogeneous encoding of relative value in their participants avoiding the heterogeneity present in our dataset. These previous datasets also highlight that our findings with regard to relative value computations are likely driven by choice or action probabilities and not a function of discount rates or self-control ability because these previous datasets show that when subjects with high discount rates are presented with choices around their indifference points, they also have positive correlations with delayed reward values in vmPFC. Such changes in the directionality of relative value computations as a function of choice or action probability represent an important target for future research.

In summary, our data provide evidence that the dlPFC supports the delay of gratification by modulating activity in a vmPFC region that reflects the stimulus value of available rewards. Our between-subjects prediction results indicate that both local activity levels and connection strengths between these brain regions mediate delay of gratification tendencies in this task. These findings also suggest that examining effective connectivity parameters in pathological populations with self-control deficits may provide useful insights into the biological basis of their dysfunction.

### Conflict of interest statement

The authors declare that the research was conducted in the absence of any commercial or financial relationships that could be construed as a potential conflict of interest.

## References

[B1] Ainslie G. (2001). Breakdown of Will. Cambridge: Cambridge University Press 10.1017/CBO9781139164191

[B2] Ballard K. Knutson B. (2009). Dissociable neural representations of future reward magnitude and delay during temporal discounting. Neuroimage 45, 143–150 10.1016/j.neuroimage.2008.11.004 19071223 PMC2685201

[B3] Balleine B. W. Daw N. O'Doherty J. (2008). Multiple forms of value learning and the function of dopamine, in Neuroeconomics: Decision-Making and the Brain, eds Glimcher P. W. Fehr E. Camerer C. Poldrack R. A. (New York, NY: Elsevier).

[B4] Bartra O. McGuire J. T. Kable J. W. (2013). The valuation system: a coordinate-based meta-analysis of BOLD fMRI experiments examining neural correlates of subjective value. Neuroimage 76, 412–427 10.1016/j.neuroimage.2013.02.063 23507394 PMC3756836

[B5] Basten U. Biele G. Heekeren H. R. Fiebach C. J. (2010). How the brain integrates costs and benefits during decision making. Proc. Natl. Acad. Sci. U.S.A. 107, 21767–21772 10.1073/pnas.0908104107 21118983 PMC3003102

[B6] Boettiger C. A. Mitchell J. M. Tavares V. C. Robertson M. Joslyn G. D'Esposito M. (2007). Immediate reward bias in humans: fronto-parietal networks and a role for the catechol-O-methyltransferase 158(Val/Val) genotype. J. Neurosci. 27, 14383–14391 10.1523/JNEUROSCI.2551-07.2007 18160646 PMC6673461

[B7] Boorman E. D. Behrens T. E. Woolrich M. W. Rushworth M. F. (2009). How green is the grass on the other side? Frontopolar cortex and the evidence in favor of alternative courses of action. Neuron 62, 733–743 10.1016/j.neuron.2009.05.014 19524531

[B8] Botvinick M. M. (2007). Conflict monitoring and decision making: reconciling two perspectives on anterior cingulate function. Cogn. Affect. Behav. Neurosci. 7, 356–366 10.3758/CABN.7.4.356 18189009

[B9] Brodersen K. H. Ong C. S. Stephan K. E. Buhmann J. M. (2010). The balanced accuracy and its posterior distribution, in 2010 20th International Conference on Pattern Recognition (ICPR) (Istanbul: IEEE), 3121–3124 10.1109/ICPR.2010.764

[B10] Carter C. S. Braver T. S. Barch D. M. Botvinick M. M. Noll D. Cohen J. D. (1998). Anterior cingulate cortex, error detection, and the online monitoring of performance. Science 280, 747–749 10.1126/science.280.5364.747 9563953

[B11] Carter R. M. Meyer J. R. Huettel S. A. (2010). Functional neuroimaging of intertemporal choice models: a review. J. Neurosci. Psychol. Econ. 3, 27–45 10.1037/a0018046

[B12] Chambers R. A. Bickel W. K. Potenza M. N. (2007). A scale-free systems theory of motivation and addiction. Neurosci. Biobehav. Rev. 31, 1017–1045 10.1016/j.neubiorev.2007.04.005 17574673 PMC2150750

[B13] Clithero J. A. Rangel A. (2013). Informatic parcellation of the network involved in the computation of subjective value. Soc. Cogn. Affect. Neurosci. [Epub ahead of print]. 10.1093/scan/nst106 23887811 PMC4158359

[B14] Clithero J. A. Smith D. V. Carter R. M. Huettel S. A. (2011). Within- and cross-participant classifiers reveal different neural coding of information. Neuroimage 56, 699–708 10.1016/j.neuroimage.2010.03.057 20347995 PMC2908207

[B15] Congdon E. Mumford J. A. Cohen J. R. Galvan A. Aron A. R. Xue G. (2010). Engagement of large-scale networks is related to individual differences in inhibitory control. Neuroimage 53, 653–663 10.1016/j.neuroimage.2010.06.062 20600962 PMC2930099

[B16] Dayan P. Niv Y. Seymour B. Daw N. D. (2006). The misbehavior of value and the discipline of the will. Neural Netw. 19, 1153–1160 10.1016/j.neunet.2006.03.002 16938432

[B17] Deichmann R. Gottfried J. A. Hutton C. Turner R. (2003). Optimized EPI for fMRI studies of the orbitofrontal cortex. Neuroimage 19(2 Pt 1), 430–441 10.1016/S1053-8119(03)00073-9 12814592

[B18] Figner B. Knoch D. Johnson E. J. Krosch A. R. Lisanby S. H. Fehr E. (2010). Lateral prefrontal cortex and self-control in intertemporal choice. Nat. Neurosci. 13, 538–539 10.1038/nn.2516 20348919

[B19] Frederick S. Loewenstein G. O'Donoghue T. (2002). Time discounting and time preference: a critical review. J. Econ. Lit. 40, 351–401 10.1257/jel.40.2.351

[B20] Friston K. J. Harrison L. Penny W. (2003). Dynamic causal modelling. Neuroimage 19, 1273–1302 10.1016/S1053-8119(03)00202-7 12948688

[B21] Gianotti L. R. Figner B. Ebstein R. P. Knoch D. (2012). Why some people discount more than others: baseline activation in the dorsal PFC mediates the link between COMT genotype and impatient choice. Front. Neurosci. 6:54 10.3389/fnins.2012.00054 22586360 PMC3345569

[B22] Green L. Myerson J. (2004). A discounting framework for choice with delayed and probabilistic rewards. Psychol. Bull. 130, 769–792 10.1037/0033-2909.130.5.769 15367080 PMC1382186

[B23] Gregorios-Pippas L. Tobler P. N. Schultz W. (2009). Short-term temporal discounting of reward value in human ventral striatum. J. Neurophysiol. 101, 1507–1523 10.1152/jn.90730.2008 19164109 PMC2666398

[B24] Hare T. A. Camerer C. F. Knoepfle D. T. Rangel A. (2010). Value computations in ventral medial prefrontal cortex during charitable decision making incorporate input from regions involved in social cognition. J. Neurosci. 30, 583–590 10.1523/JNEUROSCI.4089-09.2010 20071521 PMC6633003

[B25] Hare T. A. Camerer C. F. Rangel A. (2009). Self-control in decision-making involves modulation of the vmPFC valuation system. Science 324, 646–648 10.1126/science.1168450 19407204

[B26] Hare T. A. Malmaud J. Rangel A. (2011a). Focusing attention on the health aspects of foods changes value signals in vmPFC and improves dietary choice. J. Neurosci. 31, 11077–11087 10.1523/JNEUROSCI.6383-10.2011 21795556 PMC6623079

[B27] Hare T. A. Schultz W. Camerer C. F. O'Doherty J. P. Rangel A. (2011b). Transformation of stimulus value signals into motor commands during simple choice. Proc. Natl. Acad. Sci. U.S.A. 108, 18120–18125 10.1073/pnas.1109322108 22006321 PMC3207676

[B28] Harris A. Hare T. Rangel A. (2013). Temporally dissociable mechanisms of self-control: early attentional filtering versus late value modulation. J. Neurosci. 33, 18917–18931 10.1523/JNEUROSCI.5816-12.2013 24285897 PMC4018478

[B29] Hunt L. T. Kolling N. Soltani A. Woolrich M. W. Rushworth M. F. Behrens T. E. (2012). Mechanisms underlying cortical activity during value-guided choice. Nat. Neurosci. 15, 470–476 10.1038/nn.3017 22231429 PMC3378494

[B30] Kable J. W. Glimcher P. W. (2007). The neural correlates of subjective value during intertemporal choice. Nat. Neurosci. 10, 1625–1633 10.1038/nn2007 17982449 PMC2845395

[B31] Kable J. W. Glimcher P. W. (2010). An “as soon as possible” effect in human intertemporal decision making: behavioral evidence and neural mechanisms. J. Neurophysiol. 103, 2513–2531 10.1152/jn.00177.2009 20181737 PMC2867580

[B32] Kahnt T. Heinzle J. Park S. Q. Haynes J. D. (2011). Decoding different roles for vmPFC and dlPFC in multi-attribute decision making. Neuroimage 56, 709–715 10.1016/j.neuroimage.2010.05.058 20510371

[B33] Kasess C. H. Stephan K. E. Weissenbacher A. Pezawas L. Moser E. Windischberger C. (2010). Multi-subject analyses with dynamic causal modeling. Neuroimage 49, 3065–3074 10.1016/j.neuroimage.2009.11.037 19941963 PMC2837922

[B34] Koszegi B. Rabin M. (2006). A model of reference-dependent preferences. Q. J. Econ. 121, 1133–1165 10.1093/qje/121.4.113323607600

[B35] Lebreton M. Jorge S. Michel V. Thirion B. Pessiglione M. (2009). An automatic valuation system in the human brain: evidence from functional neuroimaging. Neuron 64, 431–439 10.1016/j.neuron.2009.09.040 19914190

[B36] Li N. Ma N. Liu Y. He X. S. Sun D. L. Fu X. M. (2013). Resting-state functional connectivity predicts impulsivity in economic decision-making. J. Neurosci. 33, 4886–4895 10.1523/JNEUROSCI.1342-12.2013 23486959 PMC6618998

[B37] Lim S. L. O'Doherty J. P. Rangel A. (2011). The decision value computations in the vmPFC and striatum use a relative value code that is guided by visual attention. J. Neurosci. 31, 13214–13223 10.1523/JNEUROSCI.1246-11.2011 21917804 PMC6623246

[B38] Luo S. Ainslie G. Pollini D. Giragosian L. Monterosso J. R. (2012). Moderators of the association between brain activation and farsighted choice. Neuroimage 59, 1469–1477 10.1016/j.neuroimage.2011.08.004 21856429 PMC12842337

[B39] McClure S. M. Ericson K. M. Laibson D. I. Loewenstein G. Cohen J. D. (2007). Time discounting for primary rewards. J. Neurosci. 27, 5796–5804 10.1523/JNEUROSCI.4246-06.2007 17522323 PMC6672764

[B40] McClure S. M. Laibson D. I. Loewenstein G. Cohen J. D. (2004). Separate neural systems value immediate and delayed monetary rewards. Science 306, 503–507 10.1126/science.1100907 15486304

[B41] McKerchar T. L. Green L. Myerson J. Pickford T. S. Hill J. C. Stout S. C. (2009). A comparison of four models of delay discounting in humans. Behav. Process. 81, 256–259 10.1016/j.beproc.2008.12.017 19150645 PMC2674118

[B42] Monterosso J. Ainslie G. (2007). The behavioral economics of will in recovery from addiction. Drug Alcohol Depend. 90, S100–S111 10.1016/j.drugalcdep.2006.09.004 17034958 PMC2756459

[B43] Monterosso J. R. Luo S. (2010). An argument against dual valuation system competition: cognitive capacities supporting future orientation mediate rather than compete with visceral motivations. J. Neurosci. Psychol. Econ. 3, 1 10.1037/a0016827 21909453 PMC3169839

[B44] Park S. Q. Kahnt T. Rieskamp J. Heekeren H. R. (2011). Neurobiology of value integration: when value impacts valuation. J. Neurosci. 31, 9307–9314 10.1523/JNEUROSCI.4973-10.2011 21697380 PMC6623498

[B46] Penny W. D. Trujillo-Barreto N. J. Friston K. J. (2005). Bayesian fMRI time series analysis with spatial priors. Neuroimage 24, 350–362 10.1016/j.neuroimage.2004.08.034 15627578

[B45] Penny W. Kiebel S. Friston K. (2003). Variational Bayesian inference for fMRI time series. Neuroimage 19, 727–741 10.1016/S1053-8119(03)00071-5 12880802

[B47] Peters J. Buchel C. (2009). Overlapping and distinct neural systems code for subjective value during intertemporal and risky decision making. J. Neurosci. 29, 15727–15734 10.1523/JNEUROSCI.3489-09.2009 20016088 PMC6666169

[B48] Peters J. Buchel C. (2010). Episodic future thinking reduces reward delay discounting through an enhancement of prefrontal-mediotemporal interactions. Neuron 66, 138–148 10.1016/j.neuron.2010.03.026 20399735

[B49] Peters J. Buchel C. (2011). The neural mechanisms of inter-temporal decision-making: understanding variability. Trends Cogn. Sci. 15, 227–239 10.1016/j.tics.2011.03.002 21497544

[B50] Plassmann H. O'Doherty J. P. Rangel A. (2010). Appetitive and aversive goal values are encoded in the medial orbitofrontal cortex at the time of decision making. J. Neurosci. 30, 10799–10808 10.1523/JNEUROSCI.0788-10.2010 20702709 PMC6634706

[B51] Pochon J. B. Riis J. Sanfey A. G. Nystrom L. E. Cohen J. D. (2008). Functional imaging of decision conflict. J. Neurosci. 28, 3468–3473 10.1523/JNEUROSCI.4195-07.2008 18367612 PMC6670602

[B52] Rachlin H. (2000). The Science of Self-control. Cambridge, MA: Harvard University Press

[B53] Rangel A. Camerer C. Montague P. R. (2008). A framework for studying the neurobiology of value-based decision making. Nat. Rev. Neurosci. 9, 545–556 10.1038/nrn2357 18545266 PMC4332708

[B54] Rangel A. Hare T. (2010). Neural computations associated with goal-directed choice. Curr. Opin. Neurobiol. 20, 262–270 10.1016/j.conb.2010.03.001 20338744

[B55] Rosa M. J. Bestmann S. Harrison L. Penny W. (2010). Bayesian model selection maps for group studies. Neuroimage 49, 217–224 10.1016/j.neuroimage.2009.08.051 19732837 PMC2791519

[B56] Shenhav A. Greene J. D. (2010). Moral judgments recruit domain-general valuation mechanisms to integrate representations of probability and magnitude. Neuron 67, 667–677 10.1016/j.neuron.2010.07.020 20797542

[B57] Stephan K. E. Penny W. D. Daunizeau J. Moran R. J. Friston K. J. (2009). Bayesian model selection for group studies. Neuroimage 46, 1004–1017 10.1016/j.neuroimage.2009.03.025 19306932 PMC2703732

[B58] Tom S. M. Fox C. R. Trepel C. Poldrack R. A. (2007). The neural basis of loss aversion in decision-making under risk. Science 315, 515–518 10.1126/science.1134239 17255512

[B59] Tzourio-Mazoyer N. Landeau B. Papathanassiou D. Crivello F. Etard O. Delcroix N. (2002). Automated anatomical labeling of activations in SPM using a macroscopic anatomical parcellation of the MNI MRI single-subject brain. Neuroimage 15, 273–289 10.1006/nimg.2001.0978 11771995

[B60] Venkatraman V. Rosati A. G. Taren A. A. Huettel S. A. (2009). Resolving response, decision, and strategic control: evidence for a functional topography in dorsomedial prefrontal cortex. J. Neurosci. 29, 13158–13164 10.1523/JNEUROSCI.2708-09.2009 19846703 PMC2801415

[B61] Volkow N. D. Wang G. J. Baler R. D. (2011). Reward, dopamine and the control of food intake: implications for obesity. Trends Cogn. Sci. 15, 37–46 10.1016/j.tics.2010.11.001 21109477 PMC3124340

[B62] Wager T. D. Sylvester C. Y. Lacey S. C. Nee D. E. Franklin M. Jonides J. (2005). Common and unique components of response inhibition revealed by fMRI. Neuroimage 27, 323–340 10.1016/j.neuroimage.2005.01.054 16019232

[B63] Wunderlich K. Rangel A. O'Doherty J. P. (2009). Neural computations underlying action-based decision making in the human brain. Proc. Natl. Acad. Sci. U.S.A. 106, 17199–17204 10.1073/pnas.0901077106 19805082 PMC2761331

[B64] Zou H. Hastie T. (2005). Regularization and variable selection via the elastic net. J. R. Stat. Soc. B Stat. Methodol. 67, 301–320 10.1111/j.1467-9868.2005.00503.x

